# Trachoma: The Last Decade?

**DOI:** 10.1080/09286586.2023.2270045

**Published:** 2023-12-12

**Authors:** Tawfik Al-Khatib, Assumpta Lucienne Bella, Martha Idalí Saboyá-Díaz, Anthony W. Solomon

**Affiliations:** a Prevention of Blindness Program, Ministry of Public Health & Population, Sana’a, Yemen; b Department of Ophthalmology, College of Medicine, University of Sana’a, Sana’a, Yemen; c Eye Unit, Al-Thawra Hospital, Sana’a, Yemen; d Faculté de Médecine et des Sciences Biomédicales, Université de Yaoundé I, Yaoundé, Cameroun; e Communicable Diseases Prevention, Control, and Elimination, Pan American Health Organization, Washington, USA; f Global Neglected Tropical Diseases Programme, World Health Organization, Geneva, Switzerland

This special issue of *Ophthalmic Epidemiology* is devoted to trachoma, the most common infectious cause of blindness. In previous such issues published by *Ophthalmic Epidemiology* over the last 8 years, guest editors have noted the momentum being generated towards global elimination of trachoma and expressed optimism for imminent success.^1–4^

Our most important message to accompany this latest trachoma special issue is the same: good progress is being made. In April 2023, an estimated 116 million people lived in areas that warrant treatment with antibiotics, facial cleanliness, and environmental improvement for trachoma elimination purposes, and an estimated 1.5 million people had trachomatous trichiasis.^5^ These estimates represent reductions of 92% and 80%, respectively, from the estimates for 2002, and reductions of 7% and 12% since June 2022.^6^ This is positive news for global health.

But we all want progress to be faster. The greater the time taken to achieve our shared targets for trachoma, the greater the number of individuals whose sight will be impaired by it. And, in the last few years, the global trachoma community has become collectively more aware of the issue of persistent and recrudescent active trachoma, realised the paucity of evidence we have to guide decisions on how to manage populations in which these phenomena are manifest, and had to reconsider its approach.^7^ Other challenges persist, too. Indigenous populations,^8^ refugees,^9,10^ internally displaced persons,^11^ populations in areas affected by conflict^12,13^ and other special groups all remain hard to reach in many places. There is also the problem of national and sub-national administrative areas that share borders across which there are substantial gradients in active trachoma prevalence; this potentially raises questions related to both the recrudescence of disease and how to undertake surveillance. To accelerate and sustain local success, we should integrate some of the knowledge and skills for delivering trachoma services into primary healthcare systems and community-based people-centred services.^14^ We have not yet generated consensus on how surveillance should be undertaken to detect post-validation recrudescence in any context – a separate issue to persistent or recrudescent active trachoma inasmuch as these things are currently defined.^7^

Another important development since publication of the last *Ophthalmic Epidemiology* trachoma special issue is the postponement of the deadline for global elimination as a public health problem: from 2020 to 2030.^15^ For political reasons, we can’t afford to have to do that again.

To that end, work continues on many fronts. As of September 2023, 18 of 62 countries known to be currently or previously trachoma-endemic had been validated as having eliminated trachoma as a public health problem; another five countries require further investigation. Trachoma prevalence estimation is robust and improving, thanks to the continuing support provided to endemic countries by Tropical Data^16^; the papers in this issue are testament to that fact, as is the confidence with which we are able to set out the estimates of global disease burden above, and to know that we have a problem from persistent and recrudescent active trachoma in some populations. Work is also ongoing to further develop model-based geostatistics^17,18^ to analyse trachoma prevalence survey output, to better understand trachoma in the Pacific Islands^19–21^ and the Torres Strait,^22^ and to consider an expanded role for conjunctival photography,^23^ amongst other efforts. Recently, WHO has launched the process of guideline development for the use of serology in trachoma elimination programmes; serology could provide a practical way to undertake integrated surveillance after the cessation of antibiotic MDA.^24–27^

Finally, partnership within the trachoma elimination community remains robust. The 24^th^ meeting of the World Health Organization Alliance for the Global Elimination of Trachoma was a face-to-face event held in Istanbul in April 2023, after several years of postponed and virtual meetings; it was well attended and invigorating. With colleagues around the globe, we look forward to overcoming the remaining hurdles to trachoma elimination and transitioning to a world in which such meetings are no longer important.

## References

[1] Mpyet C, Kello AB, Solomon AW (2015). Global elimination of trachoma by 2020: a work in progress. Ophthalmic Epidemiol.

[2] Haddad D, Nwobi B, Schmidt E, Courtright P (2016). Elimination of trachoma-knowing where to intervene. Ophthalmic Epidemiol.

[3] Engels D (2016). The global trachoma mapping project: a catalyst for progress against neglected tropical diseases. Ophthalmic Epidemiol.

[4] Cama A, Keenan JD, Dejene M, Courtright P (2018). Impact of the global trachoma mapping project. Ophthalmic Epidemiol.

[5] World Health Organization (2023). WHO alliance for the global elimination of trachoma: progress report on elimination of trachoma, 2022. Wkly Epidemiol Rec.

[6] (2003). Report of the 2nd global scientific meeting on trachoma, Geneva, 25-27 August, 2003. (WHO/PBD/GET 03.1).

[7] (2022). Informal consultation on end-game challenges for trachoma elimination, December 7–9, 2021, task force for global health, Decatur, USA.

[8] Trujillo-Trujillo J, Saboya M, Jesudason T (2022). No one left behind: how Colombia is adapting its trachoma programme to reach indigenous populations. Community Eye Health.

[9] Sanders AM, Abdalla Z, Elshafie BE, Reithinger R (2019). Prevalence of trachoma within refugee camps serving south Sudanese refugees in White Nile State, Sudan: results from population-based surveys. PLoS Negl Trop Dis.

[10] Baayenda G, Mugume F, Mubangizi A (2021). Baseline prevalence of trachoma in refugee settlements in Uganda: results of 11 population-based surveys. Ophthalmic Epidemiol.

[11] Macleod CK, Binnawi KH, Elshafie BE (2019). Unimproved water sources and open defecation are associated with active trachoma in children in internally displaced persons camps in the Darfur states of Sudan. Trans R Soc Trop Med Hyg.

[12] Yaya G (2022). Lessons learned in the implementation of programmes to eliminate trachoma within conflict zones. Trans R Soc Trop Med Hyg.

[13] Adamu MD, Mohammed Jabo A, Orji P (2022). Baseline prevalence of trachoma in 13 local government areas of Borno State, Nigeria. Ophthalmic Epidemiol.

[14] Espinal MA, Alonso M, Sereno L (2022). Sustaining communicable disease elimination efforts in the Americas in the wake of COVID-19. Lancet Reg Health Am.

[15] (2020). Ending the neglect to attain the sustainable development goals: a road map for neglected tropical diseases 2021–2030.

[16] Harding-Esch EM, Burgert-Brucker C, Jimenez C (2023). Tropical data: approach and methodology as applied to trachoma prevalence surveys. Ophthalmic Epidemiol.

[17] Amoah B, Fronterre C, Johnson O (2022). Model-based geostatistics enables more precise estimates of neglected tropical-disease prevalence in elimination settings: mapping trachoma prevalence in Ethiopia. Int J Epidemiol.

[18] Sasanami M, Amoah B, Diori AN, Ramos AN (2023). Using model-based geostatistics for assessing the elimination of trachoma. PLoS Negl Trop Dis.

[19] Butcher R, Tagabasoe J, Manemaka J (2021). Conjunctival scarring, corneal pannus and Herbert’s pits in adolescent children in trachoma-endemic populations of the Solomon Islands and Vanuatu. Clin Infect Dis.

[20] Macleod CK, Butcher R, Javati S (2021). Trachoma, anti-Pgp3 serology and ocular chlamydia trachomatis infection in Papua New Guinea. Clin Infect Dis.

[21] Lynch KD, Apadinuwe SC, Lambert SB, Nash SD (2022). A national survey integrating clinical, laboratory, and WASH data to determine the typology of trachoma in Nauru. PLoS Negl Trop Dis.

[22] Lynch KD, Brian G, Ahwang T (2022). Assessing the prevalence of trachoma: lessons from community screening with laboratory testing in Australia’s Torres Strait Islands. Ophthalmic Epidemiol.

[23] Harding-Esch EM, Naufal F, Saboya M, Jimenez C, West SK (2022). Use of Photography for Support of Trachoma Grading: Progress Report.

[24] Tedijanto C, Solomon AW, Martin DL (2023). Monitoring transmission intensity of trachoma with serology. Nat Commun.

[25] Goodhew B, Tang X, Goldstein J, Lee J, Martin D, Gwyn S (2023). Validation of immunoassays for the chlamydia trachomatis antigen Pgp3 using a chimeric monoclonal antibody. Sci Rep.

[26] Hammou J, Guagliardo SAJ, Obtel M (2022). Post-validation survey in two districts of Morocco after the elimination of trachoma as a public health problem. Am J Trop Med Hyg.

[27] Martin DL, Saboya-Diaz MI, Abashawl A, Ngondi JM (2020). The use of serology for trachoma surveillance: Current status and priorities for future investigation. PLoS Negl Trop Dis.

